# A high LDH to absolute lymphocyte count ratio in patients with DLBCL predicts for a poor intratumoral immune response and inferior survival

**DOI:** 10.18632/oncotarget.25306

**Published:** 2018-05-04

**Authors:** Colm Keane, Joshua Tobin, Dipti Talaulikar, Michael Green, Pauline Crooks, Sanjiv Jain, Maher Gandhi

**Affiliations:** ^1^ University of Queensland Diamantina Institute, Translational Research Institute, University of Queensland, Brisbane, Queensland, Australia; ^2^ Princess Alexandra Hospital, Brisbane, Queensland, Australia; ^3^ Canberra Hospital, Canberra, Australian Capital Territory, Australia; ^4^ Australian National University Medical School, Canberra, Australian Capital Territory, Australia; ^5^ MD Anderson Cancer Centre, Houston, Texas, USA; ^6^ Queensland Institute of Medical Research, Brisbane, Queensland, Australia

**Keywords:** DLBCL, tumor microenvironment

## Abstract

**Purpose:**

To test the utility of the circulating Lactate Dehydrogenase (LDH) to absolute lymphocyte count (ALC) ratio (LAR) to predict outcome to conventional first-line chemo-immunotherapy in Diffuse Large B-cell Lymphoma (DLBCL), and investigate its correlation to the tumour immune microenvironment (TME).

**Experimental Design:**

A population based cohort of 210 patients (median age: 64, range 18-90 years) with median follow up 3.8 years was analysed. All patients were treated with R-CHOP, and no immunosuppression related cases were included. Tissue for nanoString gene expression was available in 141.

**Results:**

High (i.e. adverse) LAR was associated with inferior progression free and overall survival (PFS 45% vs. 78%; OS 56% vs 86%, both p<0.001) at 5-years. Patients with a high LAR had a strikingly different TME compared to patients with a low ratio. Low LAR was associated with a good-risk TME immune gene signature (p<0.0001), including high CD8 and lower M2 macrophage infiltration. COO classification was not significantly different between high and low LAR patients. LAR was predictive of outcome independent of cell of origin and the international prognostic index (IPI). In particular, LAR discriminated patients with high IPI (3-5), showing 5-year PFS and OS of 32% vs. 74% (p=0.0006), and 43% vs. 81% (p=0.0003). A combined nanoString based immune score and the LAR allowed better prediction of outcome than either prognosticator alone (p<0.0001).

**Conclusions:**

The LAR reflects the TME within DLBCL, and is a strong predictor of outcome in DLBCL treated with conventional first-line therapy that is independent of and additive to the IPI. Further studies are required to determine if this easily applicable blood assay can determine patients that might benefit from immune checkpoint blockade.

## INTRODUCTION

Diffuse Large B-cell Lymphoma (DLBCL) is the most common aggressive lymphoma. Incorporation of rituximab to front-line chemotherapy has improved outcomes, but approximately one third of patients will relapse with subsequent poor prognosis [1–4]. The chance of cure with relapsed or refractory disease is very poor and a current clinical unmet need is for biomarkers that enables patients to be stratified for targeting with alternate agents. In particular, immune based therapies are likely to be most effective at an earlier time-point in the course of a patient's treatment [5, 6].

The International Prognostic Index (IPI) is a robust prognostic score developed over 20 years ago that is still predictive of outcome in the era of chemo-immunotherapy (e.g. ‘R-CHOP’) [7]. Some modifications to prognostic groupings have been required with the influence of the addition of rituximab to therapy but the IPI maintains its prognostic significance [8]. This score encompasses 4 clinical factors and lactose dehydrogenase as the one circulating factor. Despite its usefulness for prognostication, it does not guide therapy nor result in different strategies based on a low or high score in routine clinical practice. Patients with the poorest prognostic score still have an overall survival of greater than 50%. Better identification of patients destined to have relapsed or refractory disease would guide therapy in an era where newer agents such as targeted immunotherapies are emerging.

We have previously shown that an immune score that measures immune effectors and immune checkpoints quantified by nanoString digital multiplex gene expression in DLBCL is highly predictive and is both independent and additive to the IPI and cell of origin (COO) [9]. Immune checkpoint therapy and in particular anti-PD1 has demonstrated surprisingly robust responses in relapsed refractory Hodgkin Lymphoma [10]. Excellent responses have also been demonstrated in relapsed/refractory DLBCL [11]. It has been postulated that immune checkpoint therapy may be highly effective in certain DLBCL subtypes such as Epstein-Barr virus (EBV)-positive DLBCL and primary central nervous system lymphoma [12, 13]. It is however unclear in systemic *de-novo* DLBCL which patients may benefit from novel therapeutic approaches including checkpoint blockade. A circulating surrogate of the TME would provide important prognostic information and assist identification of patients that may benefit from risk-stratification to new therapies.

Poor prognosis is associated with a low absolute lymphocyte count and also the lymphocyte to monocyte ratio (LMR) in predicting outcome for patients with DLBCL treated with R-CHOP [14, 15]. Not only is a low lymphocyte count prognostic at diagnosis but also lymphopenia during routine follow-up after chemo- immunotherapy is a risk factor for predicting relapse [16, 17]. However, to date no simple circulating biomarker has shown useful correlations with the intratumoural environment present within the diagnostic biopsy. However, recent studies have shown that in lymphoma, LDH correlates strongly with higher levels of cell free tumour DNA and might be a surrogate of increased circulating tumour cells [18, 19]. In solid tumours such as metastatic melanoma disease bulk is directly related to LDH level [20]. In addition, there is emerging evidence that high levels of lactate in the tumour microenvironment (TME) results in significant inhibition of T cell function and is a possible mechanism of resistance to immune checkpoint therapy [21].

In metastatic melanoma treated with immune checkpoint blockade, elevated levels of LDH and low ALC counts are associated with significantly inferior responses [22]. If results are replicated in patients with DLBCL treated with conventional first-line R-CHOP chemo-immunotherapy, this will have the potential to assist selection of patients with DLBCL destined to be refractory to R-CHOP that might be candidates for checkpoint blockade within clinical trials [11]. Therefore, the aim of the current study was to investigate if the ratio of LDH to absolute lymphocyte count (LAR) at diagnosis is prognostic in patients with DLBCL treated with R-CHOP. The relationship between these circulating biomarkers and the intratumoural microenvironment was also investigated.

## RESULTS

### Patients’ characteristics

The median age of all patients (n=210) was 64.5 years (range 18-89), and the median follow-up was 3.8 years for all patients. The estimated 5-year PFS and OS was 67% and 76% respectively. The IPI score was available in 206 and was prognostic with 5-year survival for low IPI (0-2) of 87% compared to 57% for high IPI patients (IPI 3-5) (p<0.0001). COO by nanoString was available in 141 patients (66% Germinal Centre B cell DLBCL; 21% Activated B cell DLBCL; 13% unclassified) and was prognostic with 5-year survival for GCB-DLBCL of 87% compared to 53% for ABC-DLBCL patients (p=0.001).

### A high LAR score is associated with adverse prognosis

**Figure 1:**
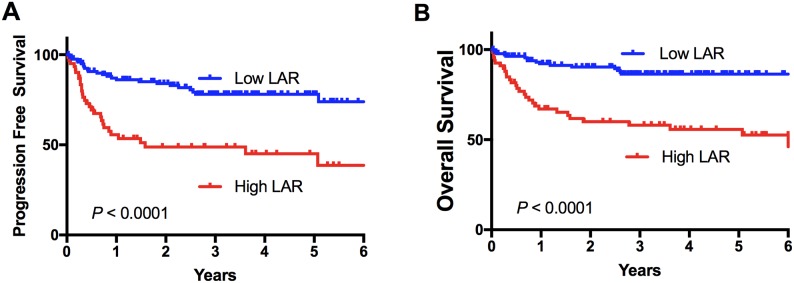
Survival stratified by the LAR score Kaplan-Meier survival curves for 210 patients stratified by the LAR score. **(A)** Progression free and **(B)** overall survival.

The median ALC was 1.2 × 10^9^/L (interquartile range [IQR] 0.76-1.89 × 10^9^/L, range 0.12-5.31 × 10^9^/L) and the median LDH count was 296 U/L (IQR 224-515 U/L, range 87-12233 U/L). A cut-off of 400 was the most discriminatory LAR value for overall survival. Using this cut-off value a high LDH to ALC ratio (LAR) score was associated with a significantly inferior PFS and OS compared to patients with a low score with 5-year PFS (45% vs 78%, HR 4.2 (95% CI 2.3-7.44, p<0.001) and OS (56% vs. 86%, HR 4.4 (95% CI 2.33-8.44, p<0.001) (see Figure 1). Of note, the LAR score was predictive of overall survival as a continuous variable (p=0.037) (Supplementary Figure 1). A high LAR score was strongly associated with 4 of the 5 components of the IPI (Table 1): not only were patients with a high LAR score (as expected) enriched for high LDH (p<0.0001), but also for higher clinical stage (p=0.0002), ECOG>1 (p=0.003), extranodal sites>1 (p=0.034). However, age >60 was not associated (p=0.65). A high LAR score was also associated with an EBV positive biopsy at diagnosis (p=0.015). For each of the individual components of the IPI (except Stage > II trend only), the LAR score was able to further stratify OS into two survival groups (Supplementary Figure 2). Of note, LAR discriminated patients with high IPI (3-5) in particular, showing 5-year PFS and OS for high and low LAR respectively of 32% vs. 74% (p=0.0006) HR 3.48(95% CI 1.75-6.75), and 43% vs. 81% (p=0.0003) HR 4.47(95% CI 2.2-9.03). There were no significant differences in the distribution of COO between the two LAR groups (p=0.09). The LAR score was however a significant predictor of outcome in the GCB subtype (92/141) but not the ABC/Unclassified groupings (49/141). Patients who had a low LAR score and were classified GCB had a 5-year OS of 98% vs. 64% HR 25.7(95% CI 7.5-88.34) with a high LAR score (p<0.0001).

**Table 1:** Association between LAR and the IPI

Patient Characteristics	LAR High (n=68)	LAR Low (n=142)	P value
Age>60	45 (66%)	88 (62%)	0.65
Stage>II	50 (75%)	64 (46%)	0.0002
ECOG>1	29 (43%)	26 (19%)	0.0003
EN>1	28 (42%)	35 (25%)	0.0345
LDH>N	58 (87%)	58 (41%)	<0.0001
IPI			
0	1 (1%)	20 (14%)	<0.0001
1,2	20 (30%)	77 (56%)	
3,4,5	46 (69%)	42 (30%)	

Full data on all the IPI individual components was available in 206 of 210 patients.

### LAR is reflective of the TME

**Figure 2:**
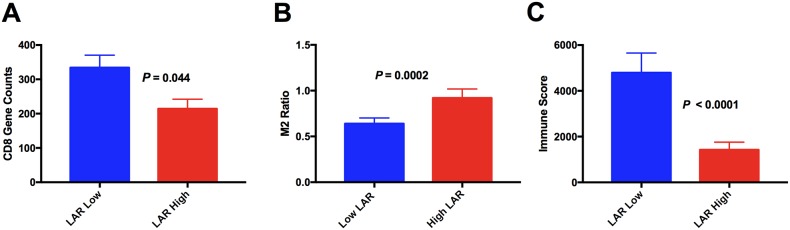
Association between LAR score and the intratumoural immune microenvironment **(A)** CD8 mRNA expression **(B)** M2 Macrophage load (derived from mRNA expression ratio of CD163/CD68) **(C)** immune score.

There were 141 patient samples with a LAR score that had sufficient tissue for digital gene expression to be performed (see Supplementary Dataset 1). The M2 count was calculated as a ratio of the CD163 to CD68 digital counts obtained. A high (adverse) LAR score was associated with significantly higher M2 macrophage count (p=0.0012) but a markedly decreased intra-tumoral CD8 count (p=0.044) (Figure 2A, 2B). This is despite the ALC on its own not predicting for the level of CD8 T cell infiltration in the TME (p=0.4). There was no difference between LAR high and low groups with regard to CD4 counts. The LAR score had a low to modest but significant positive correlation with the M2 ratio (r=0.24, p=0.003) but there was no correlation between the LAR score and CD4 or CD8 infiltration. We have previously identified a gene expression immune score derived from tumour biopsies that was highly prognostic in patients with DLBCL treated with R-CHOP, independent of IPI and gene expression based COO [9]. A good risk immune score indicates a high level of immune effectors relative to immune checkpoints, thus low scores are predictive of adverse outcome. In the current study, patients with a high LAR score had a median immune score that was 4 times lower than patients with a low LAR score (p<0.0001, Figure 2C). Patients with a good prognostic immune score were significantly more likely to have a low LAR score (p=0.0007). The LAR score showed a low to modest negative correlation with the immune score (r= -0.3, p=0.0003). These results indicate that the LAR is a circulating biomarker that partially reflects the TME within the diagnostic tumour biopsy.

### A combined immune and LAR score is highly prognostic

**Figure 3:**
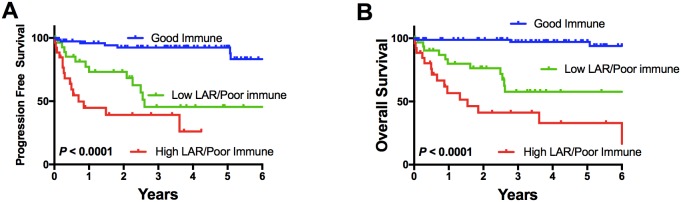
Impact of a combined immune score/LAR reflecting one group of “good immune score” alone, and the “poor prognosis” immune score patients stratified by a high or low LAR score with regard to **(A)** progression free and **(B)** overall survival.

Not only was the immune score highly prognostic but importantly a combined immune and LAR score added to the prognostic utility of either prognosticator alone, allowing prognosis to be split into three highly disparate survival groups (Figure 3). Patients with a good risk immune score had 5-year outcome of 97%. There were 61% patients in this category. Poor risk immune score patients with a low LAR score had 5-year OS of 54% (21% of patients), and those with both a poor risk immune score and a high LAR score had 5-year OS of 32% (18% of patients). This illustrates that a circulating measure of immune response provides additional information to intra-tumoral assessment of immune responses garnered from gene expression data. It also indicates that poor immune response in both the TME and the circulation is associated with very poor outcome. Interestingly, the only two deaths seen in patients with a good prognosis immune score were in patients with a high LAR score but this was not statistically significant (p=0.08).

### Multivariate survival analysis

The LAR score was included in a multivariate analysis of OS with the IPI score (there were 206 patients in whom a full multivariate analysis was possible). This showed that the LAR score was independently prognostic for PFS (P=0.001) and OS (p=0.002). In the 141 patients with nanoString based immune score and COO available, a further multivariate analysis was performed including the IPI along with the LAR score. Both the LAR score and immune Score maintained their significance independent of IPI and COO for PFS (p=0.043 and p<0.001) and OS (p=0.026 and p<0.001).

## DISCUSSION

The IPI has remained at the cornerstone of prognostication in DLBCL over the last 20 years. Despite its usefulness, it still currently provides no guidance on therapy selection. Attempts have been made to develop gene expression or IHC algorithms but to date a lack of standardisation and availability of technology have made it difficult for any biomarker derived from these methods to be used in widespread clinical practice. The present study demonstrates that an easily accessible and simple to use assay - the LAR - reflects the TME in DLBCL, and is a strong predictor of outcome in DLBCL treated with conventional first-line chemo-immunotherapy that is independent of and additive to the IPI.

The prognostic importance of LDH levels and ALC in predicting outcome to immune based therapies in solid tumours has recently been described [22]. These findings appear consistent across the subtype of immune checkpoint blockade therapies utilised to date [23]. High LDH has long been associated with poor outcome in DLBCL while the ALC has more recently been shown by us and others to be prognostic in patients with DLBCL treated with R-CHOP. It is postulated that while elevated LDH may reflect high cell turnover of aggressive disease, it has also been demonstrated that high lactate levels within the tumour bed can “stun” T cell responses [24].

We demonstrate for the first time that a high ratio of LDH to ALC was highly predictive of outcome in the setting of patients with DLBCL treated with R-CHOP. This was independent of previously described prognosticators including the IPI and COO. Strikingly, the LAR was reflective of an immune score that is generated from gene expression of the TME within the diagnostic biopsy. This immune score was on average 4-fold higher in the good prognosis low LAR patients. This data suggests a strong link between LDH and immune response to tumours. Despite this association, the LAR was still prognostically independent of this immune signature underscoring the complexity and differences of immune response between the circulation and the TME. Interestingly in the patients in whom COO was performed, the LAR score seemed to predict outcome most strongly in the GCB group. Patients who were classified GCB but had a high LAR score had a surprisingly poor outcome. We feel that the LAR score may identify the ability of the patient's immune system to clear their lymphoma. A high LAR score may reflect that a patient with a low lymphocyte count and a high tumour tumor burden is unable to mount effective long term immunologic control of their lymphoma despite chemotherapy. We anticipate that a combination of the LAR score with other immunologic, molecular and genetic prognosticators will allow new insights into the prediction of responses to emerging agents such as immune checkpoint blockade and CAR-T cell therapy.

LDH itself appears critical for tumour progression in many cancers. Tumour cells favour glycolysis over oxidative phosphorylation even in the presence of oxygen. This phenomenon, called the Warburg effect, appears critical to tumour development [25]. LDH is a critical enzyme in this process and catalyses the generation of lactate from pyruvate. Additionally, the large glucose utilization by tumours and the production of lactate has a significant role in reducing the effectiveness of immune effectors within the tumour microenvironment with consequent skewing of the TME towards one of immune suppression [21, 26]. Our data is consistent with this, with a reduced ALC being associated with higher levels of LDH. In addition, the poor prognosis high LAR ratio is strongly associated with an increase in M2 macrophages. A TME high in lactate has recently been associated with skewing of tumour associated macrophages to an immunosuppressive M2 phenotype [21]. Tumour infiltrating lymphocytes have to compete with tumour cells for glucose and high levels of lactate in the TME appear to reduce effective T cell function. It has also been postulated that high lactate levels create a more tolerogenic environment for other immune cells including dendritic cells, monocytes and NK cells [24, 25]. Indeed, evidence points to the direct targeting of LDH as a potential therapeutic avenue in cancer [26].

A number of recent studies indicate that LDH is also an effective marker of tumour bulk in solid and haematological malignancies [19, 20]. Of particular interest is a recent study describing predictors of response in patients treated with pembroluzumab in the setting of metastatic melanoma. This study demonstrated that many patients appear to demonstrate reinvigoration of the immune system with therapy but that only 50% of patients actually demonstrate clinical response despite immune activation [20]. It was demonstrated that this may be due to tumour bulk (in which LDH was found to strongly correlate in this study) which may elicit a large but ultimately futile immune response.

In summary, the LAR score is calculated from simple blood assays widely available to all physicians treating this disease. It is partially reflective of the TME, but as an independent variable also adds additional information. The prognostic importance of the LAR after conventional front-line therapy will need to be confirmed in other cohorts and in prospective studies. Patients with high LAR scores might benefit from new immune based therapies, and as with malignant melanoma, studies are required to test if LAR is a predictive biomarker of response to immune checkpoint blockade. Research into the effects of LDH and lactate on both tumour growth and immune evasion is warranted to investigate the biological basis for our findings.

## MATERIALS AND METHODS

### Patients

The study was approved by Ethics Committees at participating sites. Patients with DLBCL and treated with R-CHOP (rituximab, cyclophosphamide, doxorubicin, vincristine and prednisolone) between 2004 and 2013 were included. There were 210 patients from two participating centres. There were 120 patients from the Princess Alexandra Hospital (Queensland, Australia) and 90 patients from Canberra Hospital (Australian Capital Territory, Australia). All patients received R-CHOP, and were otherwise selected solely on the availability of clinical annotation (including survival data) and results for LDH and absolute lymphocyte count prior to initiation of therapy. The LAR score is a simple ratio of the measured LDH level over the ALC, e.g. a LDH value of 1000 U/L in a patient with an ALC of 0.5 × 10^9^/L would give a LAR ratio score of 2000 whereas a LDH level of 250 U/L with an ALC of 2.5 × 10^9^/L would give a LAR score of 100. Only *de novo* cases of DLBCL were included. Grade IIIB or transformed follicular lymphoma, HIV-positive and post-transplant patients were excluded.

### Nanostring™ nCounter RNA quantification

RNA was extracted from available formalin fixed paraffin embedded (FFPE) tumour biopsies using RecoverAll total nucleic acid extraction kit for FFPE (Ambion, Life Technologies, Carlsbad, CA, USA) as per manufacturer's instructions. The previously described immune score incorporates the gene counts of CD4^*^CD8/M2^*^PDL1, with M2 calculated by CD163/CD68(9). Genes were quantified using the nCounter platform (Nanostring™ Technologies, Seattle, WA, USA) and the COO was calculated as previously outlined [9].

### Statistical analysis

Values between groups of data were tested for statistical significance using the 2-tailed non-paired Mann-Whitney test. Categorical data were compared using Fisher's exact test or Chi-squared test as appropriate. Progression-free survival (PFS) was determined from the date of diagnosis to the date of last follow-up or disease progression, death, or discontinuation of treatment for any reason. Overall survival (OS) was determined from the date of diagnosis to the date of last follow-up or death. Survival analysis was performed using Kaplan–Meier curves and the log-rank test. Tests were two sided at p=0.05. Multivariate analysis was performed using Cox regression. Analyses were prepared using GraphPad Prism (version 6, La Jolla California USA), Statistical Package for the Social Sciences version 24 (International Business Machines Corporation, New York USA).

## SUPPLEMENTARY MATERIALS FIGURES AND TABLES




