# Supplementary material for: A high LDH to absolute lymphocyte count ratio in patients with DLBCL predicts for a poor intratumoral immune response and inferior survival
Source: Oncotarget. 2018 May 4;9(34):23620–7. doi: 10.18632/oncotarget.25306 (PMC5955090; doi:10.18632/oncotarget.25306)
Supplement: Supplementary file 1 [file oncotarget-09-23620-s001.pdf]

A high LDH to absolute lymphocyte count ratio in patients with DLBCL predicts for a poor intratumoral immune response and inferior survival

SUPPLEMENTARY MATERIALS

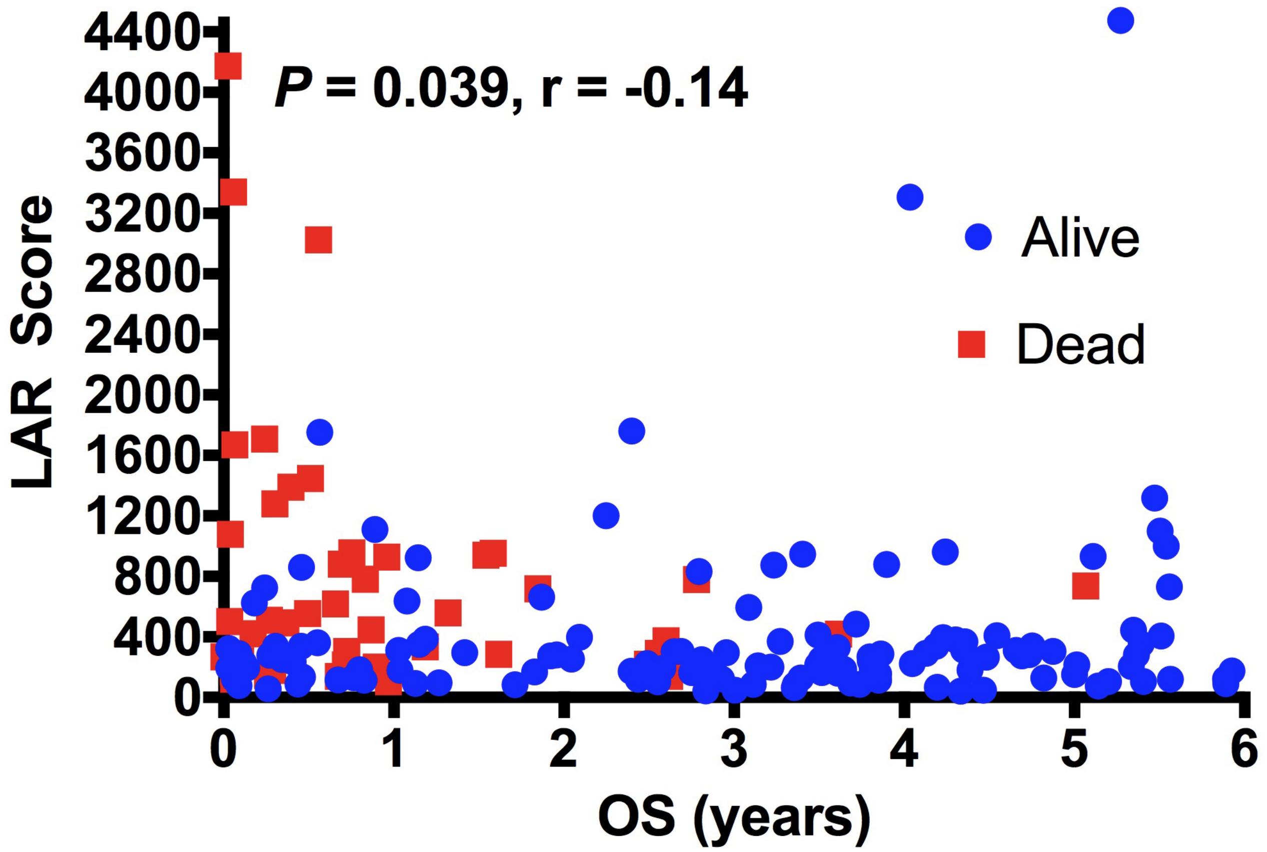

Supplementary Figure 1: Scatterplot of overall survival with LAR score as a continuous variable.

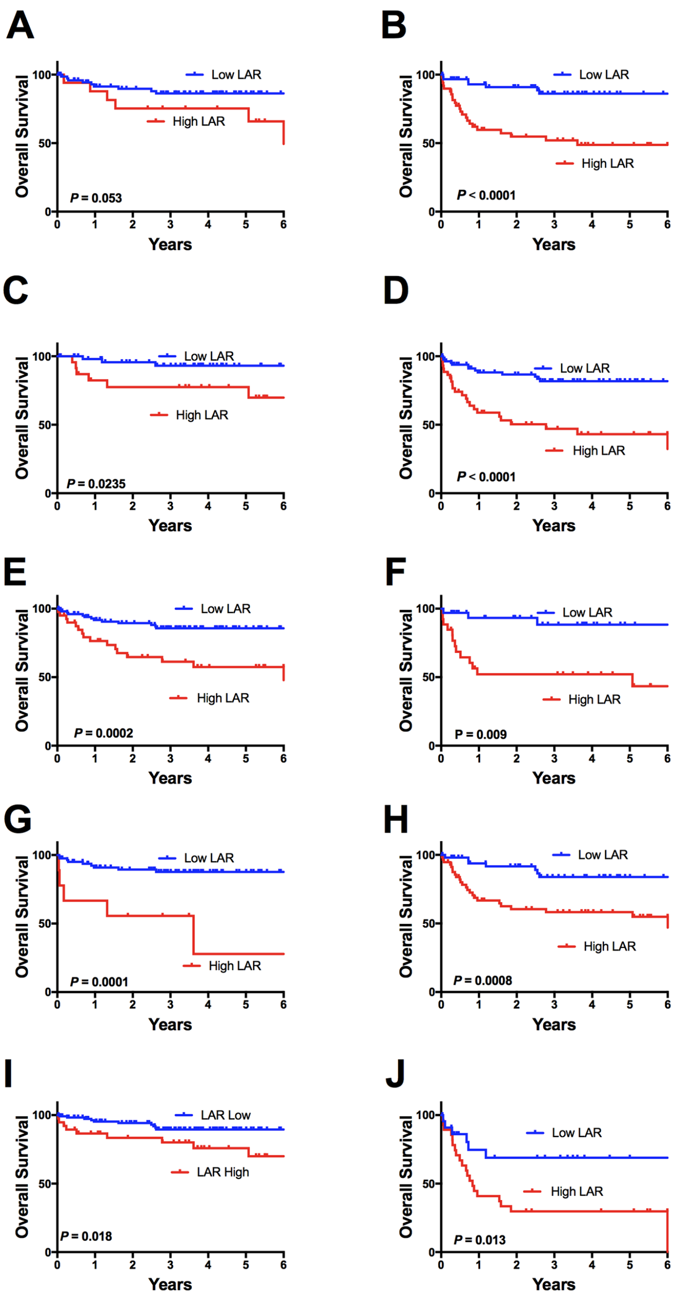

Supplementary Figure 2: Kaplan-Meier survival curves stratified by LAR and individual components of the IPI. (A) LAR score stratifying OS in patients with Stage <2. **(B)** LAR score stratifying OS in patients with Stage >2. **(C)** LAR score stratifying OS in patients with Age<60. **(D)** LAR score stratifying OS in patients with Age>60. **(E)** LAR score stratifying OS in patients with EN <1. **(F)** LAR score stratifying OS in patients with EN >1. **(G)** LAR score stratifying OS in patients with LDH < ULN. **(H)** LAR score stratifying OS in patients with LDH>ULN. **(I)** LAR score stratifying OS in patients with ECOG<1. **(J)** LAR score stratifying OS in patients with ECOG>1.

Supplementary Dataset 1: nanoString based gene expression results for genes used for analysis (normalised to GAPDH, PGAM1, PGK1, OAZ1).

See Supplementary File 1
